# Mild Steel GMA Welds Microstructural Analysis and Estimation Using Sensor Fusion and Neural Network Modeling

**DOI:** 10.3390/s21165459

**Published:** 2021-08-13

**Authors:** Leandro Bruno Alves Caio, Alysson Martins Almeida Silva, Guillermo Alvarez Bestard, Lais Soares Vieira, Guilherme Caribé de Carvalho, Sadek Crisóstomo Absi Alfaro

**Affiliations:** 1 Postgraduate Program in Mechatronic Systems (PPMEC), Campus Universitário Darcy Ribeiro, University of Brasilia, Brasilia 70910-900, DF, Brazil; laissoares.eng@gmail.com; 2 Mechanical Engineering Department, Faculty of Technology, Campus Universitário Darcy Ribeiro, University of Brasilia, Brasilia 70910-900, DF, Brazil; alyssonmartins@unb.br (A.M.A.S.); gccarval@unb.br (G.C.d.C.); sadek@unb.br (S.C.A.A.); 3 Electronic Engineering, Faculty of Gama, University of Brasilia, Gama 72405-520, DF, Brazil; guillermo@unb.br

**Keywords:** GMAW, microstructure estimation, neural networks, sensor fusion

## Abstract

This study aims at evaluating the efficiency of sensor fusion, based on neural networks, to estimate the microstructural characteristics of both the weld bead and base material in GMAW processes. The weld beads of AWS ER70S-6 wire were deposited on SAE 1020 steel plates varying welding voltage, welding speed, and wire-feed speed. The thermal behavior of the material during the process execution was analyzed using thermographic information gathered by an infrared camera. The microstructure was characterized by optical (confocal) microscopy, scanning electron microscopy, and X-ray Diffraction tests. Finally, models for estimating the weld bead microstructure were developed by fusing all the information through a neural network modeling approach. A R value of 0.99472 was observed for modelling all zones of microstructure in the same ANN using Bayesian Regularization with 17 and 15 neurons in the first and second hidden layers, respectively, with 4 training runs (which was the lowest R value among all tested configurations). The results obtained prove that RNAs can be used to assist the project of welded joints as they make it possible to estimate the extension of HAZ.

## 1. Introduction

Due to the technological development of welding processes, aspects such as the increased productivity and quality of the weld beads are of great interest to the scientific and industrial environment. Welding processes play a fundamental role in joining materials, repairing cracked components, and cladding worn parts in several areas. Among the most used metal joining processes, Gas Metal Arc-Welding (GMAW) stands out due to its high productivity and versatility, being used in several areas, for example, structural applications, manufacturing industries, and in the fabrication of devices and tools for industrial, domestic, medical, and scientific purposes—observing the limitations of dimensional precision. Also, it represents a competitive alternative to the processes with higher energy density, such as laser beam welding (LBW) and electron beam welding (EBW) [1] which motivates the various studies aimed at investigating the influence of the numerous process variables in the final microstructure [2,3,4,5]. The work performed by [2] proposed a hybrid modeling of metal bridge dynamic geometry in short-circuit GMAW for flat and orbital positions. The modeling of their work was implemented and proved to be effective in estimating the output parameters (position of the drop from the electrode tip, drop velocity, welding current, solid-state electrode length, and drop mass) providing an input data set. The research conducted by [3] a work which investigates the impact toughness behavior in high-strength low-alloy (HSLA) steels—used in naval applications—increasing heat input in coarse-grain heat-affected zone (CGHAZ). The authors pointed the essential causes of impact toughness reduction, comparing the microstructures, evaluating the extending length of fibrous cracks in cleavage fracture, calculating the local cleavage fracture stress, and applying the fundamentals of cleavage fracture mechanisms. A similar result was obtained by [4]. They identified the mechanism for reducing impact toughness in welded joints using High-Strain X80 pipeline steel based on variations in heat input and welding parameters The influence of the use of forced cooling on the tensile strength and impact resistance of Ultrahigh-strength steels was investigated by [5,6]. Using a Gleeble^®^ 3800 thermomechanical simulator, the authors in [5] varied the forced cooling finish temperature and the cooling rate and, at the end of the work, identified that better productivity can be obtained and the mechanical strength of the material can be increased using forced cooling to 100 °C to reduce the waiting time between weld passes. Gáspár [6] concluded in his work that the examined HAZ subzones in high strength steels indicated higher sensitivity to the welding heat input compared to conventional structural steels.

The microstructure of the welded joint is directly linked to its mechanical properties and also to possible defects resulting from the deposition process. Generally, the microstructure has three main regions: Fusion Zone (FZ), Heat Affected Zone (HAZ), and Base Metal (the substrate where deposition occurs). FZ is characterized by the liquid phase mixture (dilution) of the filler metal and the base metal and is generally geometrically characterized by three important parameters: reinforcement, width and depth [7,8]. The study conducted by [8] evaluated the premature Type IV cracking to clarify its mechanisms in Grade 91 steel pipe weldments using ER90S-B9 and E91T1-B9 wires. The authors observed that FZ is the region in the welded joint that presents higher strain level and hardness, lowest creep and finest grain size (martensite laths). HAZ is the portion of the base metal that was affected by heat but did not reach the melting temperature. It is composed of 4 distinct subregions named as Coarse -grained HAZ (CGHAZ), fine-grained HAZ (FGHAZ), intercritical HAZ (IHAZ), and subcritical HAZ (SHAZ).

CGHAZ corresponds to the area in which the grains of the material have grown due to high temperatures attained in welding process and maintained during a relatively long time in cooling of weld beads to room temperature. Therefore, in non-alloyed and low-carbon steels, this region is characterized by a marked increase in the size of the austenitic grain. This region is generally an area with low toughness in welded joints, as also described by [5].

In the vicinity of this subregion, the material presents a region of fine grains (FGHAZ) due to normalization processes. Unlike the boundary between FZ and CGHAZ, FGHAZ can present a combination of improved toughness and good ability to prevent crack propagation [9]. The study carried out by [9] in a C 0.053, Si 0.27, Mn 1.2, Al 0.02, (Cr+Mo+Ni) 0.42, (Nb+V+Ti) 0.082, B 0.0014, and Fe balance steel revealed that the toughness of fusion line zone is much lower than that of fine grained HAZ (FGHAZ) due its crystallographic grains. Microstructural behavior of different sub-regions of the heat-affected zone (HAZ) in steel weldment with low welding crack susceptibility. Considering the Fe-Fe_3_C phase diagram, the temperature within the CGHAZ and the FGHAZ rises above lines A3 and ACM for low carbon steels. This implies a complete austenitization of the base metal in these regions, thus significantly changing their microstructures during the cooling phase of the thermal cycle, to which both the solidifying weld pool and its vicinity are subjected during the welding process. Such an effect can be further intensified when the material is subjected to the typical GMAW process cooling rates without pre-heating or post-heating.

At a certain distance from the FZ, the material experiences a temperature range below the A3 and ACM lines, presenting IHAZ which is characterized by less severe microstructural changes and changes in the balance between the pro-eutectoid and eutectoid phases. The microstructure of IHAZ shows a mixture of fine grains formed during welding thermal cycle and coarse grains obtained from the base metal. Due to the lower temperatures reached, the region between FGHAZ and IHAZ has an irregular distribution of carbonitrides, which promotes the growth of cavities that can lead to the weld failure. The precipitation behavior of M_23_C_6_ and MX carbonitides rich in Cr and the evolution of ferritic/martesitic grains in FGHAZ from P91 welds that have Type IV failure was studied by [10]. The authors observed that the boundary between IHAZ and FGHAZ does not have a sufficiently high temperature to provide uniform distribution of the precipitates in the grains, which promotes the nucleation of the initial cavities during creep.

In the SHAZ, the temperature does not exceed the eutectoid point. At this distance, the base metal may show grain growth, subcritical annealing, or partial recrystallization. These characteristics can promote a reduction of hardness in this region of the material. Softening observed in SHAZ may also lead to strain localization and failure in the subcritical HAZ at stresses below the base material ultimate tensile strength. The welded joint microstructure can present complex phase distribution and morphology which will significantly determine the mechanical behavior of FZ and HAZ.

Problems such as fatigue cracks in the welding components can start near the HAZ (which has several microstructures derived from its welding thermal cycles) due to the high concentration of stresses in the weld joint toes. The work developed in [11] showed the characterization of the relationship between the cyclic yield properties and monotonic tensile properties of simulated HAZ microstructures. They observed that (a) most of the cyclic stress-strain relationships in HAZ, assessed using incremental step tests, agree with those of the constant stress amplitude tests, (b) the cyclic yield stresses of simulated HAZ microstructures behaved if proportional to the tensile strengths, and (c) the inclination of the proportional line in the simulated HAZ is more accentuated than that of common steels, even when the cyclical flow coefficients are proportional. The quality and functionality of weld beads produced can be associated with the weld bead geometry. However, the evaluation and control of geometrical features such as reinforcement height, width, and depth in real-time applied in arc-welding is a very complex challenge, as mentioned by [12]. The author proposed a multivariable control system in real time and also a data acquisition system for weld bead geometry based on orbital MIG/MAG welding processes. A possibility for performing these analyses in real-time is through the use of noninvasive measurement techniques such as thermography [13,14], ultrasound [15], emission spectrometry [16], and machine vision [17]. A system designed to gather the welding variables and send stimuli to the GMAW process using a constant voltage power supply was presented by [13]. This system allows the estimation of the weld bead geometry based on the fusion of thermographic data, welding current and welding voltage in a multilayer perceptron neural network. The results in this work suggest that the proposed method is applicable in industrial and research environments. The investigation of the development of a robust, low cost, point infrared sensor to monitor and control the welding process in harsh fabrication environments was performed by [14]. The sensor showed good results allowing constant depth of penetration despite the occurrence of perturbations by the feedback control of the welding process parameters. The work carried out by [15] was based on off-line and on-line measurements of noncontact ultrasound time of flight weld penetration depth. The authors pointed that this technique for measuring weld penetration depth allows the use of a closed loop to control the penetration depth of weld beads. By the relative intensity method, which is based on the Boltzmann and the Saha laws and on the definition of the emission line intensity. A non-intrusive and real-time sensor for weld defect tracking which uses emission spectrometry for measuring the electromagnetic properties of the plasma-weld pool interface in the GMA welding was developed by [16]. With the developed system, it was possible to detect grease contamination, oxidation points, slag inclusions, metal inclusion (tungsten), induced porosity, and lack of penetration. The research developed by [17] presented a real-time laser-based machine vision system to monitor and control welding processes that proved to be efficient (accuracy of (±0.55 mm). The weld bead width and height real-time measurement system, based on a high-speed camera and a long-pass optical filter mounted in a passive vision system, showed by [18] presented good results comparing to off-line data collected by a common laser-based 3D scanner. 

Algorithms of artificial intelligence (as artificial neural networks, fuzzy logic, and their associations), image processing tools and statistical techniques (i.e., multiple regression analysis, least squares, or factorial design) are widely used in the estimation of weld bead geometry. Nevertheless, the measurement and the prediction of the microstructure of weld beads are extremely difficult with current technology. Sensor fusion is based on obtaining data that cannot be acquired by direct measurements through the use of various indirect measurements provided by different sensors [19]. Taking that into account, sensor fusion techniques can be applied in the estimation of physical quantities such as the extensions of Fusion Zone and HAZ in weld beads.

Considering the current technologies of sensing, the most common method of verifying the characteristics of the microstructure of weld bead is based on post-weld manual measurements. This fact has motivated research aimed at the development of autonomous welding monitoring and control systems, based on different strategies such as sensor fusion and artificial intelligence. The work developed by [20] presents the modeling and prediction of dimensions of Heat-Affected Zone for SAW process using Finite Element Analysis (FEA) and Artificial Neural Network (ANN). The modeling was relatively accurate, although it did not distinguish between the sub-regions of HAZ. Gunaraj and Murugan [21] developed mathematical models to study the effects of welding parameters and heat input on the HAZ of submerged arc welds in structural steel pipes using. The modeling was very efficient with an accuracy of 97% in some cases. Zhang et al. [22] developed a three-dimensional (3D) finite element model to simulate the welding temperature field of a Ti-6Al-4V alloy using different welding currents based on a Gaussian heat source model. They calculated the microstructure evolution of the weld pool through the macro-micro coupling cellular automaton-finite different (CA-FD) method and obtained good results comparing with experiments carried out by GTA welding. A comparison between theoretical and experimental thermal fields as well as microstructural behavior and residual stresses applying multiple weld beads in the joint of two API 5L X52 pipe sections was conducted by [23]. The results showed that the simulation method can be used efficiently to determinate with accuracy the optimum welding parameters of this kind of weld application. The study developed by [24] aimed the application of standard multilayer feedforward networks in researches about titanium alloys. Their work includes an elaboration of TTT diagrams, determination of corrosion resistance, correlation between processing parameters, and properties of alloys, and the development of fatigue stress life diagrams for Ti-6Al-4V alloy. A study of the accuracy of a Deep Learning method for microstructural classification in the examples of certain microstructural constituents of a low carbon steel was developed by [25]. The model based in Fully Convolutional Neural Networks achieved accuracy of 93.94%. Vieira and Lambros [26] developed predictive models to estimate relations between a material’s granular microstructure and the accumulation of plastic strains at the microstructural level during plastic deformation. Physical weld simulations of single pass welding of HY 85 steel using the Gleeble^®^ 3800 thermo-mechanical simulator was performed by [27] to develop an ANN-based model for the estimation of the width and impact toughness of coarse grain heat-affected (CGHAZ) zone of simulated HAZ samples. The authors found good correlations with the backpropagation algorithm used with calculated relative error of ±3.15% for width and ±7.93% for impact toughness.

In the present work, bead-on-plate short-circuiting GMA welds had their microstructural features evaluated and associated with the welding parameters used in their production. Also, estimation models were developed to predict microstructural parameters in the FZ and the HAZ as functions of the welding parameters.

## 2. Materials and Methods

The present work presents microstructural evaluation and prediction of weld beads produced with a 1 mm diameter ESAB OK Autrod 12.51 ER70S-6 [28] wire deposited on 6.13 mm thick (6.3 mm of nominal thickness) SAE 1020 steel [29] plates, using a shielding gas composed by 96% Ar and 4% CO_2_. Three samples were produced from the deposition of weld beads on SAE 1020 steel plates 200 mm long and 50 mm wide.

Despite being a material and gas composition not commonly used in industry, and the actual thickness are different of nominal value, this research aims to evaluate the microstructure produced and the applicability of ANN in the microstructural estimation of low carbon and low alloy steels. The composition and mechanical properties [30,31] of the alloys used are shown, respectively, in Table 1 and Table 2.

The ER70S-6 wire is a manganese-silicon steel applied in the GMA welding of non-alloyed steels, such as, for example, construction steels in general with a tensile strength of about 480 MPa. Based on the data shown in Table 2, this filler metal is suitable for welding steel such as SAE 1020 and equivalents.

### 2.1. Welding Procedure

**Figure 1:**
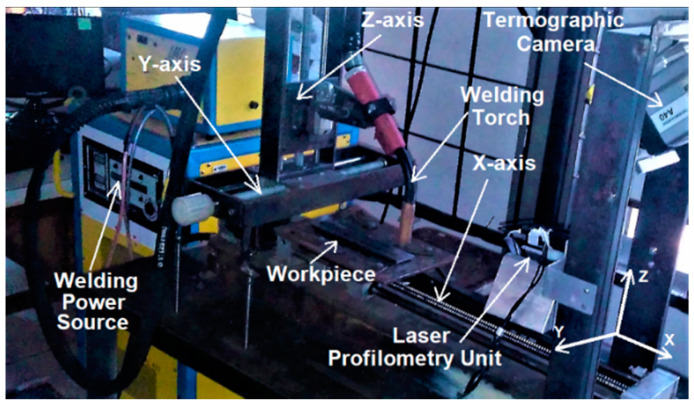
Robotic welding table with welding power source and thermographic camera.

**Figure 2:**
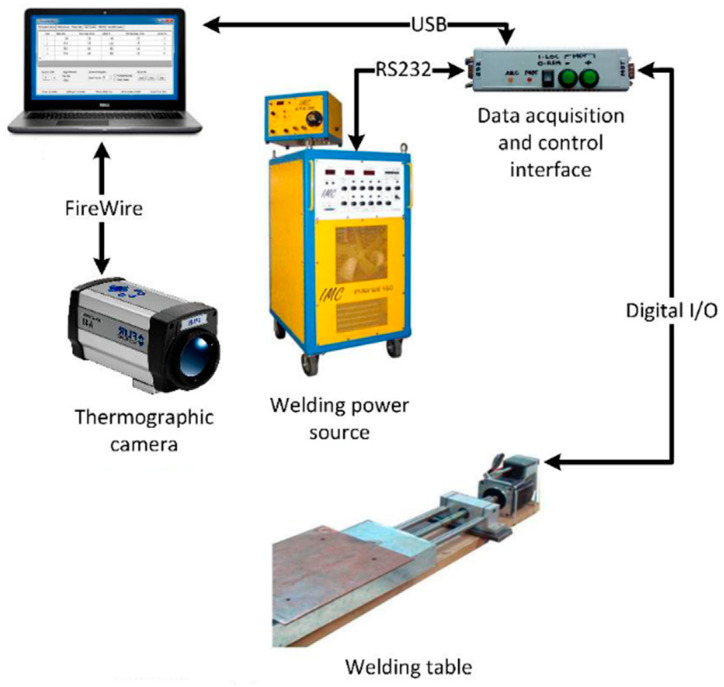
Schematic overview of components and connections of data acquisition and open-loop control system. Adapted from [13].

In the production of the weld beads, an open-loop control and data acquisition system was used. Figure 1 shows the arrangement of the equipment used in the experiments. It was composed of a welding power source, a robotic welding table, a data acquisition and control interface, a thermographic camera (Flir A40M), a laser scanning system, and a computer with data acquisition and processing software. The data acquisition, control interface, and processing software were developed in previous work [32]. Figure 2 shows a schematic overview of the components and connections of the data acquisition and open-loop control system.

The welding power source used was the Inversal 450 [33]. An algorithm developed by [32], based on RS-232 serial communication protocol, was used to establish communication with the power source in order to allow remote control and data acquisition directly from its controller. The same authors developed a state machine and a data acquisition software in Visual Studio to monitor and control the status of welding power source and the welding process operation sequence. 

The data acquisition software called Thermo Data Welding (TDW) was developed in Visual Studio by [32]. This software includes communication functions that allow for connecting to the thermographic camera through a firewire port (IEEE 1394), for both configuring the image acquisition setup and acquiring the thermal images during the process. It also connects to the welding power source, and to the robotic single-axis welding table through a specially designed interface, that connects to the computer via a USB interface and to the welding power source, through an RS-232 serial port. Using this interface, it is possible to set up welding parameters in the power source, to acquire welding data during the welding trials and to control the welding table using pulse, direction, and limit switch signals through digital IO lines (the sequence of operations for carrying out the welding trials is configured in the software before starting the welding process). Such a configuration includes: (a) the start and the final positions of the weld bead, in terms of table (workpiece) displacement; (b) the welding parameters (intensity of stimulus) sent to the welding power source (welding voltage and wire-feed speed), and (c) sampling period. The process starts manually and stops automatically when the programmed sequence ends (or when it is stopped manually). The software also allows us to configure a USB communication port on the PC, to which the data acquisition interface is connected, and to choose the data file storage location. Three files are created when the welding sequence ends: (a) a system configuration file, (b) a stimulus sequence file, and (c) a datafile with measurements collected. The measurements acquired by the interface include the following welding variables: welding speed (*w_s_*), welding voltage (*U_w_*), welding current (*I_w_*), and wire-feed speed (*w_f_*).

The robotic welding table used was developed in a previous work and it is responsible for fixing components such as the welding torch, thermographic camera and profilometry system, and for promoting the workpiece displacement. It consists of a 5 mm pitch ball screw linear axis actuated by a 3-phase stepper motor, whose resolution was adjusted to 200 steps per revolution, thus resulting in a linear resolution of 0.025 mm/step. A driver circuit controls the motor by pulses of signals that modify its speed (step time) and direction. Furthermore, other signals indicate the status of the driver and protect against overload. The maximum load supported by the structure is 147 N and the maximum travel speed is 20 mm/s. The welding speed is defined by the data sent by the computer to the data acquisition and control interface. The interface controls the driver circuit of the stepper motor in the robotic welding table.

In this way, data acquisition and control interface synchronize the welding sequence and the operation of the power source with the displacement of the workpiece, in order to obtain real-time measurements of the welding parameters. The welding voltage and current readings are performed using internal welding power source sensors. Through an RS-232 protocol serial port, the voltage and welding current information is collected by the data acquisition and control interface (with sampling period of 20 ms) and stored by the processing software. A FLIR Thermo Vision A40M camera [34] was also used in order to obtain thermographic information about weld pool. Through a Firewire (IEEE 1394) interface, the temperature of each pixel is collected in a matrix format. This model of thermographic camera uses a focal plane array (FPA) composed of 320 × 240 uncooled microbolometer detectors, sensitive to IR radiation within the spectral range between 7.5 and 13 µm, maximum temperature range from −40 °C up to 2000 °C (extended range), sampling frequency of 120 Hz, and a maximum resolution of 16-bit monochrome and 8-bit color. The camera was positioned at 45° from the horizontal plane of the workpiece and at a distance of 500 mm from the weld pool.

The next step was to cut the samples produced in the experiments with low cutting speed using a large amount of cutting fluid. This measure was taken with the objective of reducing the temperature of the samples, ensuring maximum preservation of the original microstructure.

An initial analysis of the weld beads depth was carried out using image processing techniques. After cutting, the samples were prepared for metallographic analysis by means of sanding, polishing, and etching with 5% Nital solution. 

**Figure 3:**
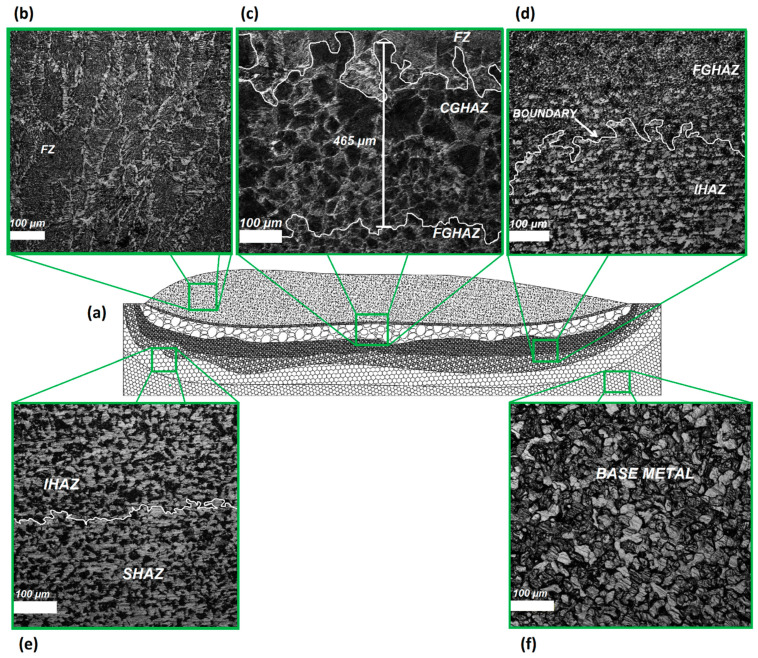
Schematic view of the location of all analyzed zones: (**a**) representation of the longitudinal section of a weld bead; (**b**) FZ; (**c**) FZ, CGHAZ, and FGHAZ boundaries highlighted using image editing tool and CGHAZ extension measurement representation (upper center); (**d**) FGHAZ/IHAZ border highlighted using image editing tool; (**e**) IHAZ/SHAZ border; (**f**) base metal.

For a better understanding of the microstructural characteristics of each sample, optical microscopy (OM), scanning electron microscopy (SEM), and X-ray diffraction (XRD) analyses were performed to confirm the phases present in the microstructure of FZ, CGHAZ, and IHAZ. Figure 3 presents a schematic view (Figure 3a) of possible regions (Figure 3b–f) that can be formed in the weld bead microstructure using ER70S-6 wire deposited on SAE 1020 steel plates. The process of identifying these zones is complex, which requires the use of optical and scanning electron microscopy techniques, in addition to the analysis of present phases. Figure 3b–d show examples of boundaries between the analyzed regions. The boundaries observed in these images were highlighted using the ImageJ™ image editor. Extension measurements were performed using the optical microscope’s measurement tool. An example of the measurement criteria used can be seen in Figure 3b.

SEM analyses were performed using a JEOL 7100 S microscope. Optical microscopy images were taken using an Olympus LEXT OLS4000 confocal laser microscope. XRD analyses were carried out using a Rigaku D/MAX-2A/C diffractometer with a Cu X-ray tube operating at 35 kV acceleration voltage and 15 mA current. The XRD analysis was conducted using the software Xpert Highscore.

In each sample produced, a single pass weld bead was deposited. The process input variables were modified according to the stimulus presented in Table 3. The purpose of this variation is to map the effect of changing process variables on the geometry and microstructure of the samples. The variable “x” indicates the distance between the tip of the wire and the start of the weld. In all experiments, the arc opens 5 mm away from the edge of the workpiece and closes at 80 mm. The change in the values of *w_s_*, *U_w_* and *w_f_* for *x* = 80 mm aimed to avoid the solidification of the pool with the wire inside at the end of the weld. The sampling period used was 20 ms.

The value of the theoretical welding current (*I_w_’*) as a function of the electrode diameter can be estimated from diagrams *wf* × *I*, for example, as is the case with the material provided in the AWS Welding Handbook [35].

With the values of *U_w_*, *I_w_*′ and as, the theoretical heat input (*H*′) can be obtained considering the thermal efficiency factor adopted by the BS ISO 1011-1 standard [36].

### 2.2. Modeling

The ANN was adopted for implementing the sensor fusion algorithm. The structure selected was the Multilayer Perceptron and Backpropagation algorithm. The input variables of the models were *U_w_*, *I_w_*, *w_f_*, *w_s_*, and five thermographic parameters (which will be explained in Section 3). For model outputs, were used 6 variants: five models with only one output (FZ, CGHAZ, FGHAZ, IHAZ, and SHAZ) and one model with all five outputs.

These combinations result in 6 network models (named with letters from A to F) with several layer configurations to estimate the extensions of analyzed zones. Models A to E consists of a microstructure prediction algorithm for FZ, CGHAZ, FGHAZ, IHAZ, and SHAZ, respectively, all with the same model structure, varying only the output. Model F represents the ANN for forecasting the five zones described above.

Each of the models was trained using two methods: Bayesian Regularization Backpropagation (BR) and Levenberg-Marquardt Backpropagation (LM). In addition, 4 retraining (training cycles from 1 to 5) were executed in each combination to refine the results in each model.

The neural network proposed for estimating the extensions of FZ and sub-regions of HAZ in weld bead was trained and validated using experimental data. Each experimental data set was divided into 70% to train, 15% to validate, and 15% to accuracy test for LM training mode. For BR training mode, the data set was divided into 70% to train and 30% to test the accuracy of the models. 

For training the ANN, a Matlab script was developed that permitted tested all possible combinations between 12 and 18 neurons in networks with 1 and 2 hidden layers, totaling 56 combinations. The input data was normalized in the Matlab neural network tool.

The results were evaluated by comparing the performance regarding the mean squared error (MSE) value presented by each network. The results for each group and all data are shown below.

## 3. Results and Discussion

### 3.1. Microstructural Evaluation

After preparation, the samples were analyzed using a confocal microscope and the mapping of the extension of FZ and subdivisions of HAZ was carried out. Measurements were taken in for all the zones every 5 mm along the longitudinal axis of the samples. The results of these measurements and the welding parameters collected by the interface, for the samples 1, 2 and 3, respectively, are shown in Table 4, Table 5 and Table 6.

Observing the values obtained for the extension of each zone in sample 1, the increase in *w_s_* and *w_f_*, both in 2x, caused a reduction in thermal input. However, there was an increase in the extension of FZ and a reduction in the extension of CGHAZ and FGHAZ.

For sample 2, the wire-feed speed was initially raised to 6 mm/s. The increase in *w_s_* and *w_f_*, both in 2x, caused a reduction in the thermal input. As a result, there was an increase in the FZ extension and a reduction in the IHAZ extension.

In sample 3, the 2x increase in *w_f_* caused an increase in thermal input and, consequently, an increase in the FZ extension and a reduction in the CGHAZ and IHAZ extensions. 

The interpretation of the data obtained in the analysis of the welding parameters can basically be based on the behavior of the heat input (*H*) when there is an increase in the wire feed speed (*w_f_*) and an increase in the welding speed (*w_s_*). The increase in *w_f_* causes an instant fluctuation in wire consumption. Thus, in order to establish the balance between fusion rate and feed rate, keeping the arc length constant, there is an increase in the electric current tending to increase the *H* value. The increase in *w_s_* causes a reduction in the *H* value, thus decreasing the penetration and the cross-sectional area of the thermally affected zone. When an increase in *w_f_* and *w_s_* occurs simultaneously, there is a combination of the two effects described above, causing changes in the *H* value in a non-linear manner.

Although the heat input has been reduced, the higher deposition rate due to the increase in the welding current tends to increase the volume and, consequently, the extension of FZ. However, less heat input provides lower penetration of the weld bead and, consequently, a lower level of metallurgical changes in the metal base, generating a reduction in the extensions of CGHAZ, FGHAZ, and IHAZ, in the case of sample 1. For sample 2, a reduction was observed only in the IHAZ. In the case of sample 3, a reduction was observed only in CGHAZ and IHAZ. Predicting which region will be affected is still a complex task that requires further studies to better understanding.

**Figure 4:**
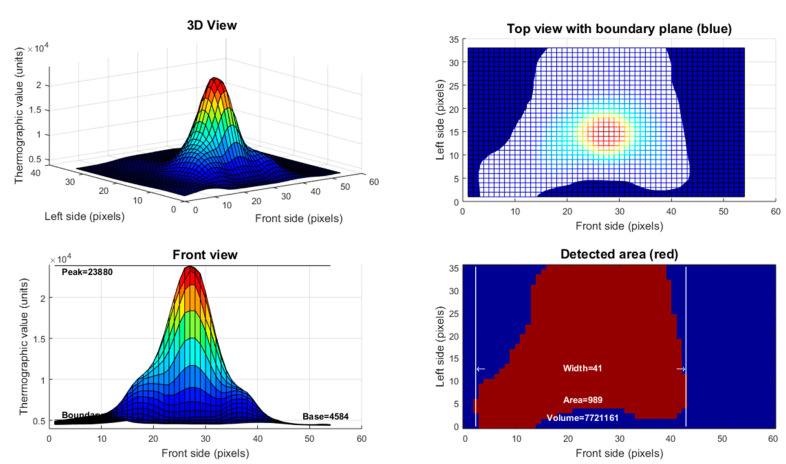
Thermal fields observed on the surface of sample 2 in thermographic value x pixels on image in three-dimensional view, top view, front view, and detected area.

During the deposition of the weld beads, information about the thermal field generated was captured. This captured information refers to a thermographic value associated with the intensity of infrared radiation emitted by different parts of the weld bead surface. The higher the temperature, the greater the intensity of the signal captured by the lens of the thermal camera. Before carrying out this analysis, it is necessary to check the emissivity of the filler metal in the liquid state so that there is no distortion in the behavior of the graphics. Thermographic data [13] was collected from the three experiments along their entire longitudinal axis. These thermographic parameters are thermographic peak (*t_p_*), thermographic base (*t_b_*), thermographic width (*t_w_*), thermographic area (*t_a_*), and thermographic volume (*t_v_*) (see Figure 4). The results were stored for use in the microstructure prediction model by ANN.

**Figure 5:**
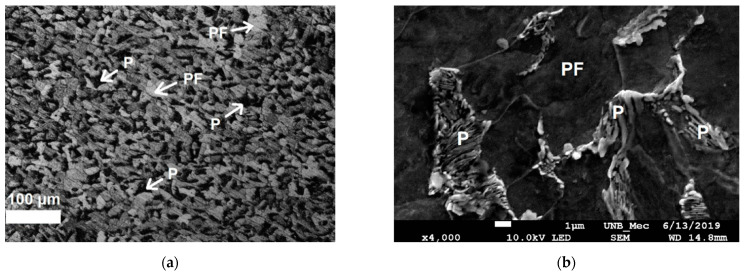
(**a**) Optical microscopy (magnification 200×) and (**b**) scanning electron microscopy (magnification 4000×) of base metal in sample 1. Etching: Nital 5%. P = Pearlite, PF = Primary Ferrite.

The results obtained in the analysis of the micrographs will be presented next. The sequence of microstructural evolution and phases present in the microstructures observed for FZ and HAZ sub-regions will be basically the same for all samples. The microstructural changes seen in the micrographs are related to the extensions of each zone considering the three samples. Therefore, the following images represent the microstructural analysis of the three samples with respect to the phase composition. The base metal microstructure is shown in Figure 5. The presence of primary ferrite (PF) and pearlite (P) is observed with a proportion of approximately 76% and 24%, respectively.

**Figure 6:**
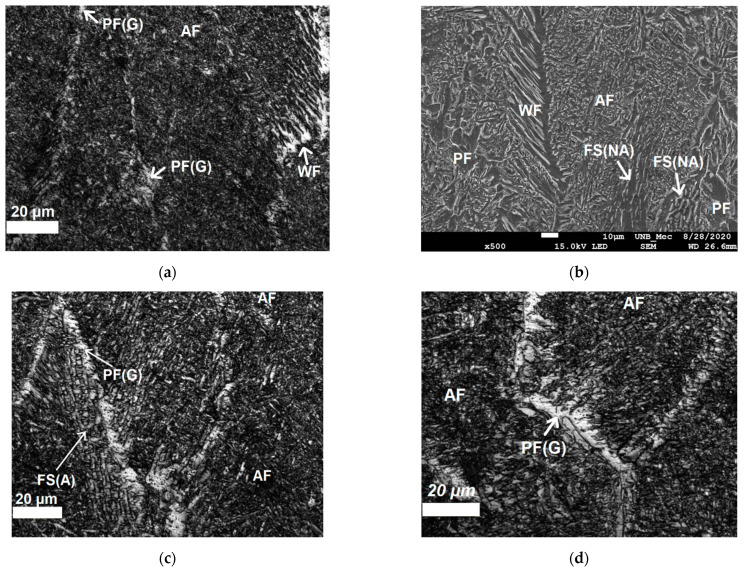
Confocal microscopy (magnification 1000×) and SEM (magnification 500×) images from microstructures of FZ in sample 1 which (**a**,**b**) show Widmanstätten Ferrite (WF) and Acicular Ferrite (AF). In (**b**) also is possible to identify presence of intragranular intragranular PF and Ferrite with Non-Aligned Second phase (FS(NA)). (**c**,**d**) show grain boundary primary ferrite (PF(G)) and AF. Etching: Nital 5%.

Figure 6a–d shows the microstructure of FZ. It is composed by grain boundary primary ferrite PF(G), acicular ferrite (AF) Widmanstätten ferrite (WF), ferrite with aligned second phase (FS(A)), and with non-aligned second phase (FS(NA)).

For many authors, the formation of bainite and Widmanstätten ferrite occurs from the same core. In this case, the nucleation process is displacive with only carbon partition during the nucleation of the new phase. The main difference between the nucleation of bainite and Widmanstätten ferrite is that the former is only formed when growth without diffusion can be supported at the transformation temperature; otherwise, Widmanstätten ferrite will form [37,38]. The basis of this model is the theory of martensitic nucleation in which the activation energy varies linearly with the chemical driving force. This principle is essentially divergent from the classical nucleation theory, in which the activation energy is inversely proportional to some chemical driving force power, considering that there are pre-existing embryos in undercooling austenite. Acicular ferrite also has a very complex nucleation mechanism, which has motivated several studies in recent years. What can be observed in this process is that the nucleation of acicular ferrite occurs heterogeneously from non-metallic inclusions in the steel [38]. Generally, in displacive nucleation, there must be a glissile interface between the nucleus and the matrix. However, it seems unlikely that the inclusion/austenite interface has this capability and dissociates directly into a ferrite embryo [37]. However, this does not preclude the displacive nucleation process. Dislocation debris from austenite adjacent to non-metallic inclusion can provide the necessary matrices (for the effects of differential thermal expansion, for example), which can dissociate into appropriate embryos. Furthermore, the activation energy and the driving force presents a linear relationship [37]. This implies that the driving force at the start temperature of transformation must vary linearly with the start temperature.

**Figure 7:**
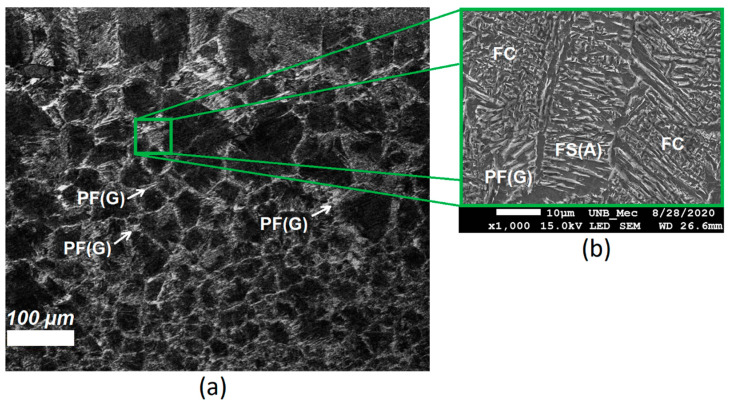
Microstructure of CGHAZ in (**a**) optical microscopy at 200× and (**b**) SEM at 1000× showing the formation of grain boundary ferrite (PF(G)), Ferrite with Aligned Second phase (FS(A)) and aggregate ferrite-carbide (FC). Etching: Nital 5%.

**Figure 8:**
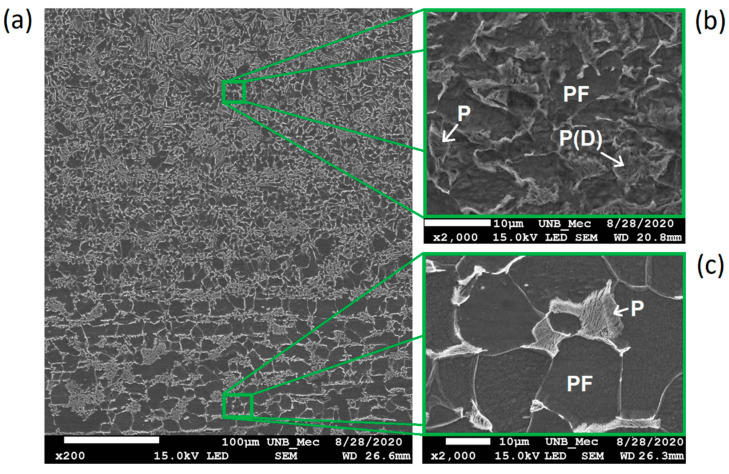
SEM images of (**a**) boundary FGHAZ/IHAZ (with 200× ampliation), (**b**) FGHAZ, and (**c**) IHAZ (2000× ampliation both). Etching: Nital 5%.

For heat affected zones, the microstructures observed are PF (polygonal and grain boundary), FS(NA), AF, and pearlite. The phase balance in HAZ remained similar to the base metal although the morphology and grain size showed variations induced by the multiple cooling rates presented by the samples with the FZ distancing. Figure 7a,b shows images of CGHAZ. Is possible to see the presence of degenerate pearlite P(D), PF(G) and aggregate ferrite-carbide (FC) in large grain colonies. Figure 8a–c shows, respectively, the microstructure of boundary FGAZ/IHAZ, FGHAZ, and IHAZ. It is possible to observe the formation of P, P(D), and PF. The microstructure formed in CGHAZ and FGHAZ has, apparently, regions of degenerate pearlite well delimited by regions of acicular ferrite (AF). This is probably due to the phase transformation of the austenite (γ) to an acicular phase. Moreover, in these parts of HAZ there might be an intense diffusional process of electrode wire alloying elements to the base metal.

**Figure 9:**
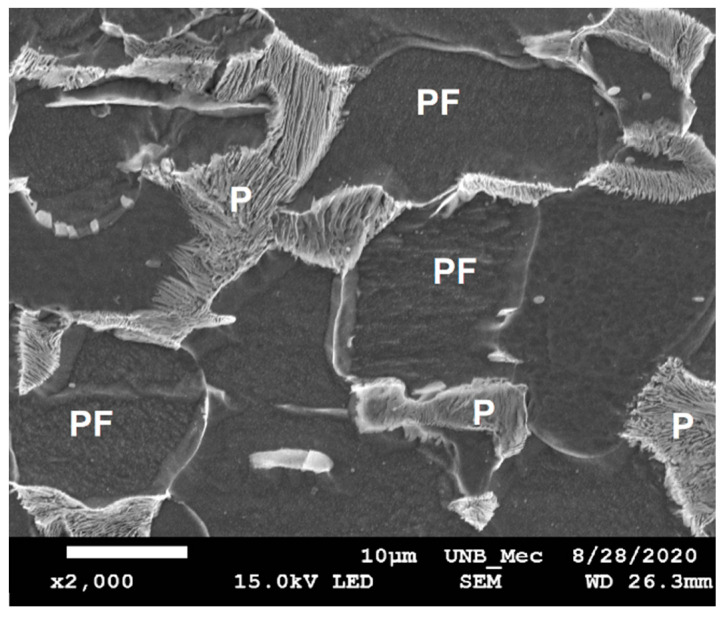
SEM image of SHAZ (2000× ampliation). Etching: Nital 5%.

Figure 9 shows the microstructure of SHAZ. It basically consists of a ferritic matrix (PF) with fine pearlite grains (P). As expected, this microstructure resembles the unaffected base metal.

**Figure 10:**
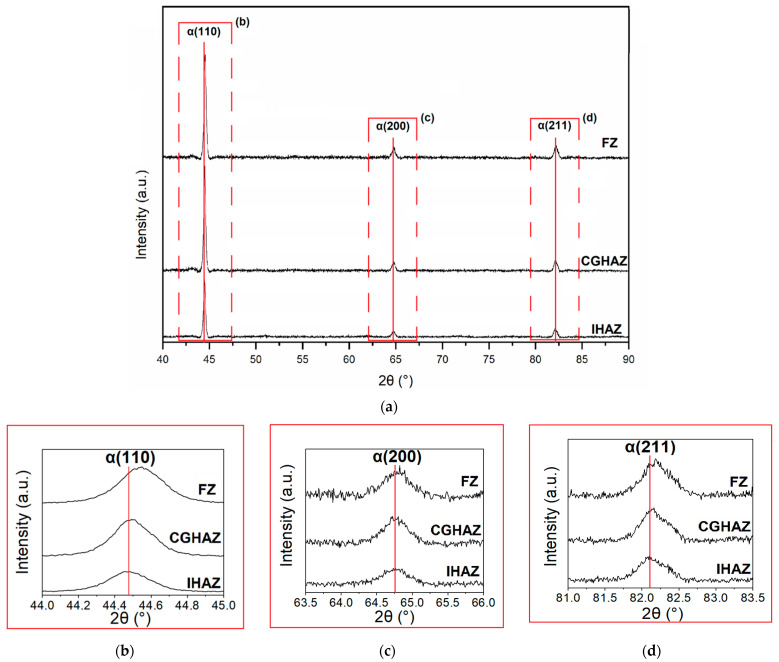
Comparison of (**a**) full XRD spectra of FZ, CGHAZ and IHAZ, (**b**) α(110) peaks, (**c**) α(200) peaks, and (**d**) α(211) peaks.

Figure 10a shows the XRD spectrum obtained for samples in three regions: FZ, CGHAZ, and IHAZ. Three diffraction peaks can be observed, which are related to the planes (110), (200), and (211) of α-Fe (JCPDS 89-7194). The obtained spectrum indicates that no residual austenite was found, as observed in the microscopy analysis. However, as shown in Figure 10b–d, in comparison with CGHAZ and IHAZ, the diffraction signal of FZ showed greater intensity and width for the 3 peaks (2θ equal to 44.5°, 64.75°, and 82.07°, respectively). Furthermore, although there are no obvious changes in the intensities of α (200) and α (211), the intensity of the α (110) peak is significantly increased from the IHAZ region to CGHAZ and reaches its highest value for FZ. This is the result of the refinement of the grain presented by the microstructures of FZ and CGHAZ, since they present the occurrence of AF. In general, the widening of the FWHM results from a decrease in grain size and an increase in dislocation density.

According to the relationship established by Bragg’s law, increasing 2θ angle indicates an increase in the spacing of a pair of adjacent network planes, suggesting that the α-Fe phase network parameter was changed due to the increase in the content of solid solution elements throughout the α-Fe matrix during the deposition of the filler metal in FZ. Also, dislocations of the peaks can be associated to oxides formation in FZ.

### 3.2. ANN Results

**Figure 11:**
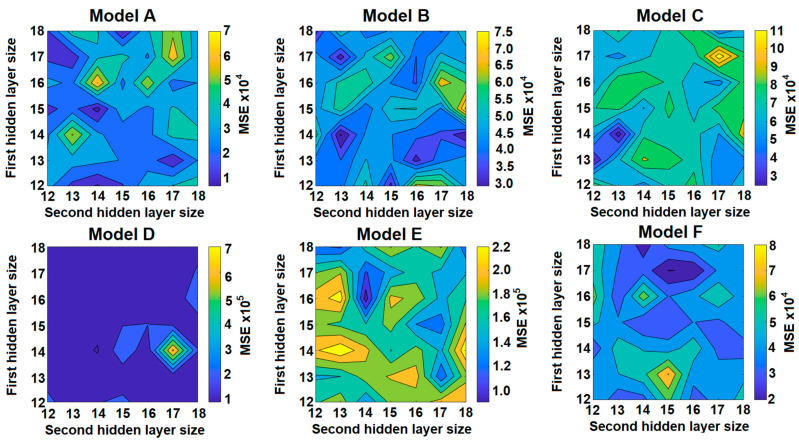
ANN with best results (lower MSE value) in each combination of the size of hidden layers, changing the training method and training cycles. The figure shows the lowest MSE obtained in models A to F for each association between the number of neurons in the first and second hidden layers.

A comparison of the best MSE value for each layer size combination is shown in Figure 11) through diagrams that relate number of neurons in the first and second hidden layers and the best MSE results (considering all tests performed varying the mode and number of training cycles). Darker shades on the diagrams represent lower MSE values. Through the diagrams it is possible to notice that each model has different ranges of MSE. Model A (ZF extension as an output) had the smallest MSE.

In addition to the MSE, the square root of the MSE, or Root of the Mean Square Error (RMSE), is commonly used to express the accuracy of the numerical results with the advantage that RMSE presents error values in the same dimensions of the analyzed variable. 

Table 7 shows a simplified comparison between the best network results for each combination of number of neurons in the hidden layers for all models.

The RMSE values obtained demonstrate that the modeling has a relevant error range in Models D and E, for respectively IHAZ and SHAZ. However, observing the extensions of CGHAZ and FGHAZ, it is possible to classify the models B and C as the most critical due to the relevancy of RMSE value. This was already expected since the formation of these regions occurs according to a chaotic and non-linear process of microstructural formation.

The lowest RMSE value was obtained for model A–FZ as output—which uses 15 and 14 neurons in the first and second hidden layers, respectively. This hidden layer combination showed lower RMSE for BR training mode and 2 training runs configuration. 

**Figure 12:**
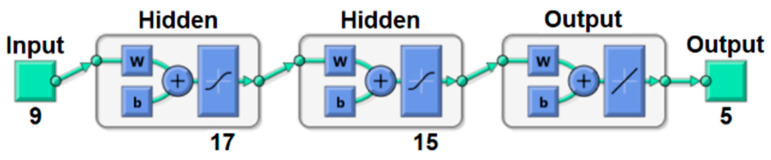
ANN structure of model F.

However, model F–5 zones as outputs showed good fit with 17 and 15 neurons in the first and second hidden layers, respectively, and with BR training mode and 4 training runs. A scheme of the structure of model F is shown in Figure 12. It has 9 input variables (*U_w_*, *I_w_*, *w_s_*, *w_s_*, *t_p_*, *t_b_*, *t_w_*, *t_a_*, and *t_v_*), 17 and 15 neurons in first and second hidden layers, respectively, and 5 outputs (FZ, CGHAZ, FGHAZ, IHAZ, and SHAZ extensions).

**Figure 13:**
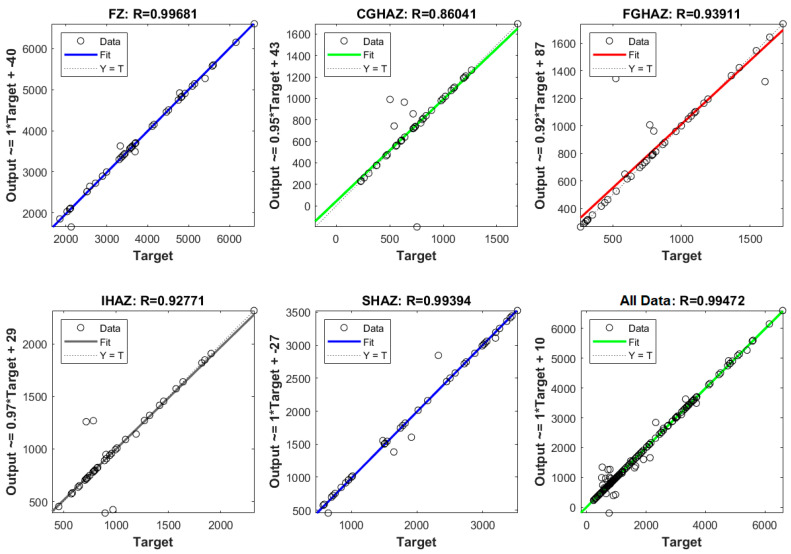
Comparison of linear regressions of targets by variable relative to outputs in model F.

Figure 13 shows linear regressions of each of the 5 outputs of model F and a regression of all data in this model. “All Data” refers to a linear regression with all results of the outputs of model F (FZ, CGHAZ, FGHAZ, IHAZ, and SHAZ). The F model is more advantageous than other single output models because it integrates the 5 outputs or model layers in a single model but with a similar structure. This model requires similar computing resources to estimate all 5 layers as individual models to estimate only one layer. Another significant judgment to select this model is the R value (0.99472), which is better than CGHAZ (0.86041), FGHAZ (0.93911), IHAZ (0.92771), and SHAZ (0.99394), surpassed only by FZ (0.99681).

Table 8 presents a comparison between the Overall Performance average, standard deviation, minimum, maximum, and amplitude of RMSE values for all models from A to F.

The results found indicate that the model with lower RMSE values is Model A (except or the standard deviation, which presented the second lower RMSE). The standard deviation of all models indicates that there is a high dispersion for the RMSE values. This dispersion might be associated to the variations of precision in output estimation in the algorithms when the configurations of 1st and 2nd hidden layer sizes changes. This fact highlights the relevance of the results founds for the network configurations with lower RMSE value. Table 9 shows the average errors relative to each zone for Model F. The final error of the model is about 12.9%, which represents a relatively good fit for estimation of zone extents. However, model F can estimate the extent of FZ with an error of only 4% which represents a satisfactory result.

Some authors have conducted research using ANN to estimate the geometry [39,40,41] and the mechanical properties [42,43] of weld beads in various welding processes, achieving an error range consistent with the results presented in this study. However, as it is a little explored topic, there is not a large number of studies that correlate the prediction of the extension of the regions present in the material’s microstructure with ANN. Thus, for the material used in this work, there are no publications to comparisons between the results.

## 4. Conclusions

At the end of this work, the following conclusions can be pointed out:

- Increasing the wire speed causes an increase in the FZ extension. The increase in FZ tends to reduce the extension of CGHAZ, as was observed in all 3 samples.
- The microstructural analysis revealed that there is no presence of residual austenite in the samples. The predominant phases in FZ are AF, PF (G), and WF, as expected for the used material. Initially, the presence of AF, PF, and P (D) at CGHAZ was verified. As the thermally affected zone moves away from the material, the fractions of AF and PD decrease as grains of P begin to appear and the presence of PF increases. In the IHAZ and SHAZ regions, only the presence of PF and P was found, which corroborates with the microstructure initially presented in base metal. The displacement in the diffraction peaks in XRD analyses indicates a possible increase in the lattice constant comparing FZ with IHAZ due to the higher content of Mn and Si. The displacements of diffraction peaks may also be associated with the formation of oxides.
- Model A with 15 and 14 neurons in the 1st and 2nd hidden layers, using Bayesian Regularization and 2 training loops, showed the lowest MSE value among all models. Nevertheless, model F can be considered the most satisfactory results for zone extent estimation. Model F gives all 5 zones as outputs and showed a good result (R = 0.99472) with 17 and 15 neurons in the 1st and 2nd hidden layers. In addition, it becomes more convenient to deal with a model that provides all 5 outputs instead of using 5 individual models for estimating the microstructure of the welded region.
- It is important to note that the modelling presented here is valid for the welding conditions established in the methodology of this work (composition of the alloys, substrate dimensions, wire diameter, composition of the shielding gas, tension and measurement interval). In addition, the applied methodology is valid for analyses of microstructural evolution in the direction perpendicular to that of travel speed.
- The F model presents an overall error of approximately 12.9%, which is a satisfactory result considering the modelling complexity that involved different regions of the microstructure. Furthermore, this presented an error of 4% in the modelling of the ZF extension, which represents a great adjustment of the adopted estimation model.
- The ANN modelling presented in this work presented an error relatively similar to that of some works in which the geometry and mechanical properties of the beads were estimated. Furthermore, no work involving the modeling of the extent of regions in FZ and HAZ in weld beads by ANN was found in the literature.
- The use of the sensor fusion algorithm, presented in this work, to processing the thermographic information and welding arc variables allows a better understanding of microstructural characteristics of the welded joint and can help in designing and dimensioning welded structures. The results show that the modelling methodology to estimate the microstructure of the welded region, using the implementation of sensor fusion with ANN, is a valuable result to material area research and to process control.

## Figures and Tables

**Table 1:** Chemical compositions of wire and base metal [30,31].

Alloy	Chemical Composition Limits (%)				
	C	Mn	P (Max)	S (Max)	Si
OK Autrod 12.51 ER70S-6	0.08	1.50	-	-	0.90
SAE 1020	0.18 to 0.23	0.30 to 0.60	0.30	0.50	-

**Table 2:** Mechanical properties of wire and base metal [30,31].

Alloy	Yeld Strength (Mpa)	Tensile Strength (Mpa)	Elongation (%)	Charpy V Toughnesss (J)	Hardness (HB)
OK Autrod 12.51 ER70S-6	470	560	26	90 (at −20 °C)	-
SAE 1020	250	475	20	-	140

**Table 3:** Welding parameters used in samples 1, 2, and 3.

Sample	*x* (mm)	*w_s_* (mm/s)	*U_w_* (V)	*w_f_* (m/min)	*I_w_*′ (A)	*H*′ (J/mm)
1	5	6	30	4	122	488
	30	12	30	8	207	414
	55	6	30	4	122	488
	80	10	20	3	0	0
2	5	6	30	6	160	640
	30	12	30	12	280	560
	55	6	30	6	160	640
	80	10	20	3	0	0
3	5	6	30	6	160	640
	30	6	30	12	280	1120
	55	6	30	6	160	640
	80	10	20	3	0	0

**Table 4:** Measurements corresponding to sample 1.

*x* (mm)	Sample 1 Measurements									
	Process Inputs					Process Outputs				
	*w_s_* (mm/s)	*U_w_* (V)	*I_w_* (A)	*w_f_* (m/min)	Heat Input (J/mm)	FZ (μm)	CGHAZ (μm)	FGHAZ (μm)	IHAZ (μm)	SHAZ (μm)
5	6.0	29.8	130.8	4.0	555.3	2515	556	442	703	2502
10	6.0	29.9	144.9	4.0	570.8	1855	1094	960	793	3011
15	6.0	29.9	150.2	4.0	521.2	2092	1195	794	939	2448
20	6.0	30.0	139.2	4.0	557.7	2520	1188	789	818	2015
25	10.3	30.0	137.0	5.1	308.8	2585	718	799	904	2324
30	12.0	29.5	204.1	8.0	389.1	3385	378	303	583	2989
35	12.0	30.0	209.1	8.0	377.7	3347	260	291	714	3033
40	12.0	30.8	206.1	8.0	373.9	2996	232	314	1320	2578
45	12.0	30.0	202.6	8.0	379.7	2725	303	265	720	3524
50	7.7	29.9	204.9	5.1	600.4	2031	1020	1000	779	3416
55	6.0	29.9	133.3	4.0	527.9	2110	979	714	889	3363
60	6.0	30.0	137.0	4.0	516.7	2115	228	1094	1091	3194
65	6.0	30.0	133.6	4.0	557.8	2131	754	522	970	3192

**Table 5:** Measurements corresponding to sample 2.

*x* (mm)	Sample 2 Measurements									
	Process Inputs					Process Outputs				
	*w_s_* (mm/s)	*U_w_* (V)	*I_w_* (A)	*w_f_* (m/min)	Heat Input (J/mm)	FZ (μm)	CGHAZ (μm)	FGHAZ (μm)	IHAZ (μ m)	SHAZ (μm)
5	6.0	28.7	173.7	6.0	664.7	3685	465	318	573	2746
10	6.0	29.8	176.6	6.0	701.7	3706	611	733	453	2877
15	6.0	29.9	185.6	6.0	739.9	3691	502	605	783	1647
20	6.0	30.0	183.6	6.0	734.4	4907	723	463	1009	3442
25	6.0	30.0	172.2	6.0	688.8	6151	476	416	994	3254
30	12.0	29.8	235.1	12.0	467.1	4812	607	525	958	1511
35	12.0	30.0	250.3	12.0	500.6	4777	540	588	715	1479
40	12.0	29.9	249.7	12.0	497.7	4454	375	879	749	1502
45	12.0	29.9	252.2	12.0	502.7	3598	610	352	789	2717
50	12.0	29.9	249.8	12.0	497.9	3301	611	1050	825	1544
55	6.0	30.0	169.1	6.0	676.4	3627	718	747	800	1783
60	6.0	30.0	173.7	6.0	694.8	3445	640	864	941	2160
65	6.0	29.9	180.4	6.0	719.2	3329	635	770	1191	1914
70	6.0	30.0	180.8	6.0	723.2	2895	733	785	1415	1513

**Table 6:** Measurements corresponding to sample 3.

*x* (mm)	Sample 3 Measurements									
	Process Inputs					Process Outputs				
	*w_s_* (mm/s)	*U_w_* (V)	*I_w_* (A)	*w_f_* (m/min)	Heat Input (J/mm)	FZ (μm)	CGHAZ (μm)	FGHAZ (μm)	IHAZ (μm)	SHAZ (μm)
5	6.0	29.7	166.9	6.0	554.9	4109	564	633	649	3060
10	6.0	30.0	169.3	6.0	650.6	4152	1019	1164	1271	1819
15	6.0	29.9	187.7	6.0	652.6	4507	1209	1546	1847	955
20	6.0	30.0	165.3	6.0	610.2	5087	1074	1364	1819	914
25	6.0	29.3	177.4	6.0	646.2	4833	1695	813	1639	1000
30	6.0	30.1	234.8	10.3	681.6	6600	739	1104	1454	754
35	6.0	29.3	246.5	12.0	839.5	5601	889	1364	730	1017
40	6.0	29.3	237.9	12.0	887.3	5141	814	1741	637	846
45	6.0	29.0	258.3	12.0	885.8	5399	790	1610	895	649
50	6.0	29.3	244.9	12.0	906.2	5574	804	1194	908	724
55	6.0	29.9	174.1	7.7	897.2	4747	839	1644	800	698
60	6.0	30.0	173.9	6.0	649.3	3701	995	1422	719	959
65	6.0	29.9	177.3	6.0	657.9	3561	1264	697	2317	589
70	6.0	30.0	172.6	6.0	660.8	3586	1104	1071	1908	573
75	6.0	30.0	178.7	6.0	645.8	3421	603	312	1573	1744

**Table 7:** Measurements corresponding to sample 1.

Model	Zone	Hidden Layer 1	Hidden Layer 2	Training Mode	Training Cycles	MSE (μm^2^)	RMSE (μm)
A	FZ	15	14	BR	2	6499	81
B	CGHAZ	14	13	BR	4	29,395	171
C	FGHAZ	14	13	LM	1	25,007	158
D	IHAZ	14	14	LM	2	91,116	302
E	SHAZ	16	14	LM	3	90,131	300
F	ALL	17	15	BR	4	19,660	140

**Table 8:** Comparison between the standard deviation for average, minimum and maximum MSE values for all models from A to F.

ANN Model	A	B	C	D	E	F
Average MSE Overall Performance (μm)	328.63	367.42	421.90	781.02	786.77	481.66
Standard Deviation (μm)	342.05	325.58	477.49	777.17	725.95	564.80
Minimum MSE (μm)	80.62	171.46	158.11	301.83	300.17	140.36
Maximum MSE (μm)	1113.55	1174.73	1962.14	2385.37	2224.86	2076.05
MSE Amplitude (μm)	1113.55	1187.43	1967.23	2404.16	2244.99	2078.46

**Table 9:** Comparison between the average errors relative to each zone for Model F.

Region	Sample 1 Error	Sample 2 Error	Sample 3 Error	Average Sample Error
FZ	5.6%	3.5%	3.0%	4.0%
CGHAZ	20.4%	23.8%	14.6%	19.6%
FGHAZ	22.0%	22.0%	11.9%	18.6%
IHAZ	16.2%	16.8%	11.0%	14.7%
SHAZ	4.8%	5.6%	12.9%	7.8%
Average microstructural error	13.8%	14.3%	10.7%	12.9%
